# Effect of marker choice and thermal cycling protocol on zooplankton DNA metabarcoding studies

**DOI:** 10.1002/ece3.2667

**Published:** 2017-01-12

**Authors:** Laurence J. Clarke, Jason M. Beard, Kerrie M. Swadling, Bruce E. Deagle

**Affiliations:** ^1^ Antarctic Climate & Ecosystems Cooperative Research CentreUniversity of TasmaniaHobartTas.Australia; ^2^ Australian Antarctic DivisionKingstonTas.Australia; ^3^ Institute for Marine and Antarctic StudiesUniversity of TasmaniaHobartTas.Australia

**Keywords:** cytochrome oxidase subunit I, environmental DNA, metabarcoding, mitochondrial 16S ribosomal DNA, nuclear 18S rDNA, zooplankton

## Abstract

DNA metabarcoding is a promising approach for rapidly surveying biodiversity and is likely to become an important tool for measuring ecosystem responses to environmental change. Metabarcoding markers need sufficient taxonomic coverage to detect groups of interest, sufficient sequence divergence to resolve species, and will ideally indicate relative abundance of taxa present. We characterized zooplankton assemblages with three different metabarcoding markers (nuclear 18S rDNA, mitochondrial COI, and mitochondrial 16S rDNA) to compare their performance in terms of taxonomic coverage, taxonomic resolution, and correspondence between morphology‐ and DNA‐based identification. COI amplicons sequenced on separate runs showed that operational taxonomic units representing >0.1% of reads per sample were highly reproducible, although slightly more taxa were detected using a lower annealing temperature. Mitochondrial COI and nuclear 18S showed similar taxonomic coverage across zooplankton phyla. However, mitochondrial COI resolved up to threefold more taxa to species compared to 18S. All markers revealed similar patterns of beta‐diversity, although different taxa were identified as the greatest contributors to these patterns for 18S. For calanoid copepod families, all markers displayed a positive relationship between biomass and sequence reads, although the relationship was typically strongest for 18S. The use of COI for metabarcoding has been questioned due to lack of conserved primer‐binding sites. However, our results show the taxonomic coverage and resolution provided by degenerate COI primers, combined with a comparatively well‐developed reference sequence database, make them valuable metabarcoding markers for biodiversity assessment.

## 1 Introduction

Recent research has begun to validate metabarcoding as a time and cost‐efficient method for biodiversity surveys in terrestrial, freshwater, and marine ecosystems (Hirai, Kuriyama, Ichikawa, Hidaka, & Tsuda, 2015; Ji et al., 2013; Thomsen et al., 2012; Valentini et al., 2016). The results of metabarcoding studies depend on the markers used providing sufficient taxonomic coverage and resolution for the taxa of interest. The coverage of metabarcoding markers is more of an issue for taxonomically diverse samples such as zooplankton surveys that include a wide range of metazoan and nonmetazoan phyla. However, greater taxonomic coverage often comes at the cost of taxonomic resolution.

Conserved markers such as those targeting nuclear 18S ribosomal DNA (rDNA) provide broad taxonomic coverage across the eukaryotic domain of life (Lindeque, Parry, Harmer, Somerfield, & Atkinson, 2013), but provide limited taxonomic resolution compared to markers targeting mitochondrial cytochrome oxidase *c* subunit I (COI, Tang et al., 2012). COI markers can also take advantage of “barcode” databases (Hebert, Cywinska, Ball, & deWaard, 2003; Ratnasingham & Hebert, 2007). However, as COI is a protein‐coding gene, “third codon wobble” increases the chance of primer mismatches when targeting genetically diverse taxonomic groups. Indeed, the lack of conserved primer‐binding sites has been shown to cause taxonomic bias for many COI markers (Clarke, Soubrier, Weyrich, & Cooper, 2014; Piñol, Mir, Gomez‐Polo, & Agusti, 2015). Mitochondrial 12S and 16S rDNA has been proposed as an alternative source of metabarcoding markers (Clarke et al., 2014; Deagle, Jarman, Coissac, Pompanon, & Taberlet, 2014; Epp et al., 2012) to avoid taxonomic bias introduced by primer‐template mismatches but retain taxonomic resolution. Most zooplankton metabarcoding studies to date have targeted nuclear 18S (Chain, Brown, MacIsaac, & Cristescu, 2016; Lindeque et al., 2013; Mohrbeck, Raupach, Martinez Arbizu, Knebelsberger, & Laakmann, 2015; Pearman, El‐Sherbiny, Lanzén, Al‐Aidaroos, & Irigoien, 2014; Sun et al., 2015) or 28S rDNA (Hirai et al., 2015). Zhan, Bailey, Heath, and MacIsaac (2014) compared the performance of mitochondrial COI, 16S, and nuclear 18S markers for metabarcoding zooplankton, but were unable to generate high‐quality PCR products for COI with four different primer sets, and recommended 18S over mitochondrial 16S based on broader taxonomic coverage. Although not applied directly to zooplankton, Leray and Knowlton (2015) and Leray et al. (2013) used a new COI primer set to characterize marine benthic communities and fish diet, highlighting its potential for assessing marine metazoan biodiversity.

The “holy grail” of metabarcoding is to retrieve relative abundance data through the proportion of reads assigned to each taxon. Many studies have highlighted the potential of metabarcoding as at least a semiquantitative method for both nuclear ribosomal (Hirai et al., 2015; Lindeque et al., 2013; Sun et al., 2015; Weber & Pawlowski, 2013) and mitochondrial DNA markers (Evans et al., 2016; Kelly, Port, Yamahara, & Crowder, 2014; Murray et al., 2011). Biases introduced during DNA extraction, PCR amplification, and sequencing are likely to skew the number of reads per taxon, with a disproportionate effect of primer‐template mismatches on PCR‐amplification efficiency (Elbrecht & Leese, 2015; Piñol et al., 2015). Hence, it may be particularly difficult to retrieve relative abundance data targeting protein‐coding genes such as COI. However, a study using environmental DNA metabarcoding to characterize aquatic mesocosms found “mismatch potential” for six mitochondrial primer sets had no consistent effect on the relationship between species biomass and high‐throughput sequencing (HTS) read abundance (Evans et al., 2016).

In this study, we compared the performance of one nuclear (18S) and two mitochondrial (COI and 16S rDNA) metabarcoding markers for characterizing zooplankton assemblages from Storm Bay, Tasmania. Southeast Australia is a global marine “hotspot” (Hobday & Pecl, 2013), with the greatest projected increases in sea surface temperature predicted to occur northeast and east of Tasmania (Lough, Gupta, & Hobday, 2012), including Storm Bay. Two of the markers (COI and 18S) have previously been used to characterize taxonomically diverse marine samples (Leray et al., 2013; Zhan et al., 2013, 2014); the third was designed to amplify mitochondrial 16S rDNA from calanoid copepods, one of the most abundant and diverse components of the zooplankton. To compare performance of the three markers, we evaluated taxonomic coverage and resolution, correspondence between morphology‐ and DNA‐based identification, and the ability to assess relative abundance of calanoid copepods from the proportion of HTS reads. For the COI marker, high annealing temperatures in the first rounds of the published touchdown PCR protocol (Leray et al., 2013) could bias PCR amplification toward taxa with less mismatches in the primer‐binding sites (Sipos et al., 2007); hence, we compared the number of taxa detected using the touchdown protocol to a protocol with a single low annealing temperature. We also explored the technical repeatability of taxon detection by re‐sequencing COI amplicons generated with identical PCR protocols.

## 2 Materials and Methods

### 2.1 Sample collection

**Figure 1:**
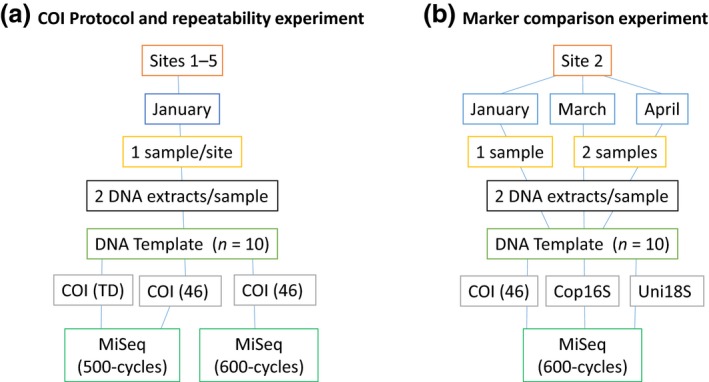
Flow chart of experimental design for testing (a) repeatability of taxon detection and (b) marker comparison. 46—annealing temperature = 46°C, TD—touchdown protocol

For DNA‐based identification, zooplankton samples were collected from five sites in Storm Bay, Tasmania on 27 January 2015 (one sample per site). A weighted bongo net (750 mm diameter, 200 μm mesh), equipped with a flow meter, was lowered to within 5 m of the sea floor and towed vertically through the water column at approximately 1 m/s. Two biological replicate samples were also collected at one of the sites on 10 March and 22 April 2015. Separate samples were collected at the same times for morphological identification. January samples were used to explore the repeatability of operational taxonomic unit (OTU) detection with the COI marker, while samples from site 2 (January, March and April) were used to compare the performance of the three metabarcoding markers against morphological identification methods (Figure 1). Samples were preserved in 70% ethanol at 4°C prior to DNA extraction or in 10% buffered formalin prior to morphological identification. For morphological identification, the three samples collected at site 2 were split using a Folsom Plankton Splitter to obtain approximately 400–600 zooplankton per sample. Zooplankton were identified to the lowest practical taxon based on morphology and counted.

### 2.2 DNA extraction

Two technical replicates from each sample, consisting of 2 ml of plankton, were centrifuged at 3,000 rpm for 1 min and the supernatant removed. The tissue was homogenized with a Bio‐Gen PRO200 tissue homogenizer (PRO Scientific, Oxford, CT, USA) for 30 s at the lowest speed (5,000 rpm). The homogenate was centrifuged at 850 x g for 1 min, and 10–40 mg tissue transferred to a new tube. DNA was extracted using the QIAGEN DNeasy Blood & Tissue kit (QIAGEN, Doncaster, Vic., Australia) by adding 180 μl buffer ATL to the tissue, and following the manufacturer's instructions, incorporating an overnight lysis at 56°C. Extracts were eluted in 2 × 100 μl buffer EB and stored at −20°C. No template controls were extracted and stored in the same manner.

### 2.3 PCR amplification and high‐throughput sequencing

#### 2.3.1 Effect of thermal cycling protocol for COI

We tested whether the PCR protocol altered the taxa detected with the COI marker by amplifying the five January samples (10 extracts) with two different thermal cycling protocols. PCR amplifications were performed in two rounds, the first to amplify the target locus and add sample‐specific 6 bp multiplex‐identifier (MID) tags (forward and reverse primer) and Illumina sequencing primers, the second to add sequencing adapters and additional 10 bp MIDs (Table 1). The first round was either (A) the touchdown protocol as per Leray et al. (2013), namely 94°C for 10 min, a 16 cycle touchdown phase (62–1°C per cycle), followed by 25 cycles with an annealing temperature of 46°C (total of 41 cycles), and a final extension at 72°C for 5 min, or (B) the same protocol using 35 cycles with a single annealing temperature (46°C). Three replicate first round PCRs were performed for each DNA extract with each thermal cycling protocol. Each reaction mix contained 2 mM MgCl_2_, 200 μM dNTPs, 0.5 μM each of forward and reverse primer, 2 μg BSA, 0.5 U AmpliTaq Gold DNA polymerase in 1 × reaction buffer (Life Technologies, Melbourne, Australia), and 1 μl DNA extract (undiluted or 1:10 dilution) in a total reaction volume of 10 μl. The optimum quantity of template DNA was determined with qPCR for each extract (Murray, Coghlan, & Bunce, 2015). Replicate PCR products were pooled then diluted 1:10 and Illumina sequencing adapters added in a second round of PCR (10 cycles with an annealing temperature of 55°C) using the same conditions as the first round, except primer and MgCl_2_ concentration was reduced to 0.1 μM each and 1.5 mM, respectively. Products from each round of PCR were separated by electrophoresis and visualized on 2% agarose gels. Second round PCR products were pooled in equal ratios based on band intensity (Murray et al., 2015). The pooled products were purified using Agencourt AMPure XP beads (Beckman Coulter, Brea, CA, USA) and the size distribution and concentration of the library assessed on a 2100 Bioanalyzer (Agilent Technologies, Santa Clara, CA, USA). The pool was diluted to 2 nM and paired‐end reads generated on a MiSeq (Illumina, San Diego, CA, USA) with MiSeq Reagent Nano Kit v2 (2 × 250 bp).

**Table 1:** PCR primers used in this study (first and second round). The position of multiplex identifiers (MIDs) is shown by “X”. Amplicon lengths are based on OTUs from this study and exclude primer sequences. bp—base pairs

Primer name	Sequence (5′–3′)	Locus	Length (bp)	References
First round primers				
ILF_Cop16SF	TCGTCGGCAGCGTCAGATGTGTATAAGAGACAGXXXXXX TAAGGTAGCATARTAATTWG	Mitochondrial 16S rDNA	315 ± 36	This study
ILR_Cop16SR	GTCTCGTGGGCTCGGAGATGTGTATAAGAGACAGXXXXXX TAATTCAACATCGAGGTC			
ILF_mlCOIintF	TCGTCGGCAGCGTCAGATGTGTATAAGAGACAGXXXXXX GGWACWGGWTGAACWGTWTAYCCYCC	Mitochondrial COI	313 ± 10	Leray et al. (2013)
ILR_jgHCO2198	GTCTCGTGGGCTCGGAGATGTGTATAAGAGACAGXXXXXX TAIACYTCIGGRTGICCRAARAAYCA			
ILF_Uni18S	TCGTCGGCAGCGTCAGATGTGTATAAGAGACAGXXXXXX AGGGCAAKYCTGGTGCCAGC	Nuclear 18S rDNA	419 ± 26	Zhan et al. (2013)
ILR_Uni18SR	GTCTCGTGGGCTCGGAGATGTGTATAAGAGACAGXXXXXX GRCGGTATCTRATCGYCTT			
Second round primers				
msqF	AATGATACGGCGACCACCGAGATCTACACXXXXXXXXXX TCGTCGGCAGCGTCAGATGTGTATAAGAGACAG			
msqR	CAAGCAGAAGACGGCATACGAGATXXXXXXXXXX GTCTCGTGGGCTCGGAGATGTGTATAAGAGACAG			

To explore repeatability of PCR amplification and sequencing, PCR amplicons were generated a second time using the 35 × 46°C protocol as described above and sequenced on a MiSeq using the MiSeq Reagent Kit v3 (2 × 300 bp) with the amplicon libraries for the marker comparison.

#### 2.3.2 Comparing metabarcoding markers

DNA extracts for January, March, and April, including no template controls, were PCR‐amplified with two existing and one newly designed primer set (Table 1). We used ecoPrimer (Riaz et al., 2011) to design new primers (Cop16SF and Cop16SR) based on 33 mitochondrial genomes from 11 copepod species. The ecoPrimer parameters used were a maximum of three mismatches between each primer and the target sequence with no mismatches allowed within two nucleotides of the 3′ end. Amplicon length was restricted to 100–600 bp. Primers were required to have no mismatches in at least 50% of species (option −q 0.50), with no more than three mismatches in at least 60% of species (−s 0.60). Primer design was refined based on calanoid copepod 16S sequences in Geneious version 8.1.7 (http://www.geneious.com, Kearse et al., 2012).

PCR amplifications were performed in two rounds as described above. Three replicate PCRs were performed with each marker for each DNA extract. Uni18S and Cop16S markers were amplified with Phusion DNA polymerase (New England Biolabs, Ipswich, MA, USA), with each reaction mix containing 0.1 μM (Uni18S) or 0.3 μM (Cop16S) each of forward and reverse primer, 2 μg bovine serum albumin (BSA), 0.2 U Phusion DNA polymerase in 1 × Phusion Master Mix (New England Biolabs), and 1 μl DNA extract (undiluted or 1:10 dilution) in a total reaction volume of 10 μl. PCR thermal cycling conditions were initial denaturation at 98°C for 30 s, followed by 30 cycles of 98°C for 5 s, 53°C (Uni18S) or 45°C (Cop16S) for 20 s, and 72°C for 20 s, with a final extension at 72°C for 5 min. COI could not be amplified with Phusion polymerase due to inosine residues in the reverse primer and was amplified with AmpliTaq Gold (Life Technologies, Melbourne, Australia), using 35 cycles with an annealing temperature of 46°C as described above. Replicate PCR products were pooled then diluted 1:10 and Illumina sequencing adapters added in a second round of PCR (10 cycles with an annealing temperature of 55°C) using the same conditions as the first round, except primer concentration was reduced to 0.1 μM each and MgCl_2_ concentration was reduced to 1.5 mM for COI. Products from each round of PCR were separated by electrophoresis and visualized on 2% agarose gels. Pooling and purification were performed as described above, with paired‐end sequencing performed on a MiSeq using MiSeq Reagent Kit v3 (2 × 300 bp).

### 2.4 Data analysis

Reads were deconvoluted based on 10 bp MIDs on the MiSeq. Fastq reads were merged using the ‐fastq_mergepairs command in USEARCH v8.0.1623 (Edgar, 2010). Merged reads were sorted by “internal” 6 bp MID tags, and locus‐specific primers trimmed with custom R scripts using the *ShortRead* package (Morgan et al., 2009), with only reads containing perfect matches to the expected MIDs and primers retained. Reads for all samples were dereplicated and global singletons discarded (‐derep_fulllength ‐minuniquesize 2), and clustered into OTUs with the UPARSE algorithm (Edgar, 2013) at either the default 97% identity (Cop16S and COI, ‐otu_radius_pct 3) or 99% identity (Uni18S, ‐otu_radius_pct 1) using the “‐cluster_otus “ command. Potentially chimeric reads were also discarded during this step. Reads for each sample were then assigned to OTUs (‐usearch_global ‐id 0.97 for Cop16S and COI, ‐id 0.99 for Uni18S), and an OTU table generated using a custom R script. Although clustering 18S reads into OTUs may prevent distinct taxa from being detected (Brown, Chain, Zhan, MacIsaac, & Cristescu, 2016), no additional taxa were detected based on the nonclustered 18S reads (data not shown). A paired *t*‐test was used to compare the number of OTUs and taxa per site in January detected with COI using either the touchdown thermal cycling protocol or the 46°C protocol.

Taxonomy was assigned to each OTU using MEGAN version 5.10.5 (Huson, Mitra, Ruscheweyh, Weber, & Schuster, 2011) based on 50 hits per OTU generated by BLASTN searches against the NCBI “nt” database excluding environmental sequences (downloaded June 2016). The lowest common ancestor (LCA) algorithm used to assign taxonomy in MEGAN only assigns an OTU to species (or other taxonomic level) if no other species (or taxon) has a blast hit within a specified percentage of the score of the best hit (top percent parameter). The same LCA parameters were used for the three markers (default parameters, except Min support = 1, Min score = 300, Top percent = 10), except that top percent = 5 for Uni18S as this provided better agreement with the morphology‐based taxonomy. A bit score of 300 is equivalent to *ca*. 80% identity with 100% query coverage for each marker. OTUs assigned to species by the LCA algorithm were inspected, with species‐assignment only retained if the identity was >95%.

#### 2.4.1 Beta‐diversity

Morphology‐based counts and HTS read counts for site 2 were fourth‐root‐transformed. Differentiation among collection months for each metabarcoding marker was compared using Bray–Curtis distance based on a rarefied OTU table and visualized using principle coordinate analysis plots generated with QIIME v1.8.0 (beta_diversity_through_plots.py, Caporaso et al., 2010), with strength and significance of groupings assessed using the Adonis method (compare_categories.py, 999 permutations). The taxa or OTUs contributing to the difference between months were identified using SIMPER analysis based on the fourth‐root‐transformed OTU tables and morphology‐based counts with the *vegan* package (Oksanen et al., 2015) in R version 3.2.1 (R Core Team 2015). The significance of an OTU's contribution was estimated using a permutation approach (999 permutations).

#### 2.4.2 Correlation of calanoid copepod HTS reads and biomass

We compared the strength of the correlation between biomass (determined from morphological species counts) and number of HTS reads per taxon for the three metabarcoding markers. We focused on calanoid copepods as all markers were capable of PCR‐amplifying this group and the biomass of each species could be estimated from counts. Counts for each sex of each species were converted to dry weights (μg) based on sex‐specific prosome lengths from the literature using the approach of Hirai et al. (2015). HTS read counts and dry weights were summarized at family‐level and converted to the percentage of total calanoid HTS reads or dry weight. Pearson (*r*) and Spearman rank (ρ) correlation coefficients were calculated for correlations between the proportions of HTS reads and biomass using the “cor.test” function in R (R Core Team 2015).

## 3 Results

### 3.1 Effect of thermal cycling protocol on taxon detection

We compared OTUs detected with the COI marker from amplicons generated with either (1) the published touchdown PCR protocol (Leray et al., 2013) or (2) a single annealing temperature (46°C) and a reduced number of cycles (amplicons sequenced on the same MiSeq run). Despite similar numbers of reads per sample (mean ± SD = 14,700 ± 4,000 and 15,600 ± 2,800 for 46°C and touchdown protocols, respectively), 254 OTUs representing 105 taxa were detected from the January samples using the 46°C annealing temperature, compared to only 200 OTUs (96 taxa) using the touchdown protocol (Table 2). The number of OTUs per site was significantly less using the touchdown protocol (paired two‐tailed *t‐*test, *t *=* *13.04, *df* = 4, *p *=* *.0002), although the number of taxa detected per site was not significantly different (*t *=* *2.16, *df* = 4, *p *=* *.097). As the primers are designed to target metazoans, it is possible the lower annealing temperature would decrease PCR specificity, leading to detection of additional nonmetazoan taxa. However, detection of both metazoan and nonmetazoan taxa was increased using the single 46°C annealing temperature, albeit with a larger proportional increase in nonmetazoans (Table 2).

**Table 2:** Comparison of OTUs and taxonomic assignments for a COI marker PCR‐amplified using either a touchdown thermal cycling protocol (Leray et al., 2013) or 35 cycles using a single annealing temperature (46°C). Zooplankton were collected in January 2015 from five sites in Storm Bay, Tasmania. Taxonomic assignments were performed using MEGAN 5 based on BLASTN searches against the NCBI “nt” database (downloaded June 2016)

	46	Touchdown
No. OTUs	254	200
No. taxa	105	96
Metazoan taxa	75	73
Nonmetazoan taxa	30	23

### 3.2 Repeatability of COI amplification & sequencing

The COI marker was PCR‐amplified from the January DNA extracts (annealing temperature = 46°C) on two occasions and sequenced on separate MiSeq runs, allowing us to assess repeatability of the PCR and sequencing for this marker. One replicate was excluded due to low coverage (Jan 1B, firstrun, 126 reads vs. 2,320–22,900 reads for other replicates). Despite using different chemistries (v2 and v3) and numbers of cycles (2 × 250 and 2 × 300) in the two sequencing runs, OTUs representing more than 1% of reads in one replicate were always detected in the corresponding replicate PCR. Similarly, OTUs representing more than 0.1% of reads in at least one PCR replicate were typically detected in both replicates (97.1%).

### 3.3 Comparison of three metabarcoding markers and morphological ID

**Figure 2:**
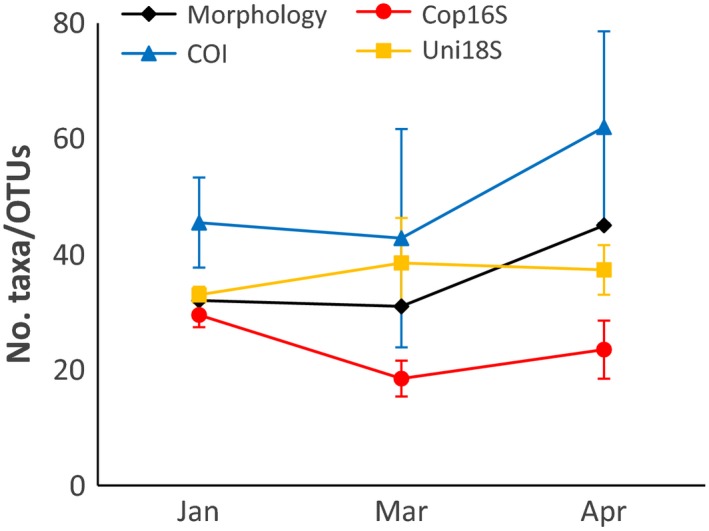
Changes in number of operational taxonomic units (OTUs) for metabarcoding markers and number of morphologically identified taxa in Storm Bay, Tasmania, between January and April 2015. Values for metabarcoding markers are means ± *SD*

No template controls produced a small number of merged HTS reads (2–124) for each marker; however, no reads were retained for these samples after discarding sequences with mismatches in the primer or MIDs. The total number of reads with no mismatches to the expected MID and primers for the DNA extracts from the site sampled in January, March, and April (site 2, *n* = 10 samples; including 2 biological replicates in March and April and 2 extracts for each collection) ranged from 47227 (COI) to 98870 (Uni18S), with rarefaction plots approaching a plateau for each marker (Figure S1 in Appendix). The number of OTUs detected was two‐ to threefold higher for COI compared to Uni18S and Cop16S (Table 3). The number of OTUs detected per month was most similar to the number of morphologically identified taxa for Uni18S, with COI showing an increase from March to April, reflecting the increase in morphologically identified taxa in this time period (Figure 2).

**Table 3:** Summary of taxonomic assignments for Storm Bay zooplankton communities from site 2 based on HTS data for three genetic markers. Taxonomic assignments performed using MEGAN 5 based on BLASTN searches against NCBI “nt” database (downloaded June 2016)

	Cop16S	COI	Uni18S
No. OTUs	62	181	97
Unassigned OTUs	20 (32.3%)	61 (33.7%)	2 (2.1%)
No. phyla (zooplankton phyla)	5 (5)	18 (9)	11 (10)
No. species (zooplankton species)	13 (13)	29 (26)	10 (8)
Crustacea			
No. orders	4	6	3
No. families	11	13	10
No. species	10	16	3

#### 3.3.1 Taxonomic coverage and resolution

**Figure 3:**
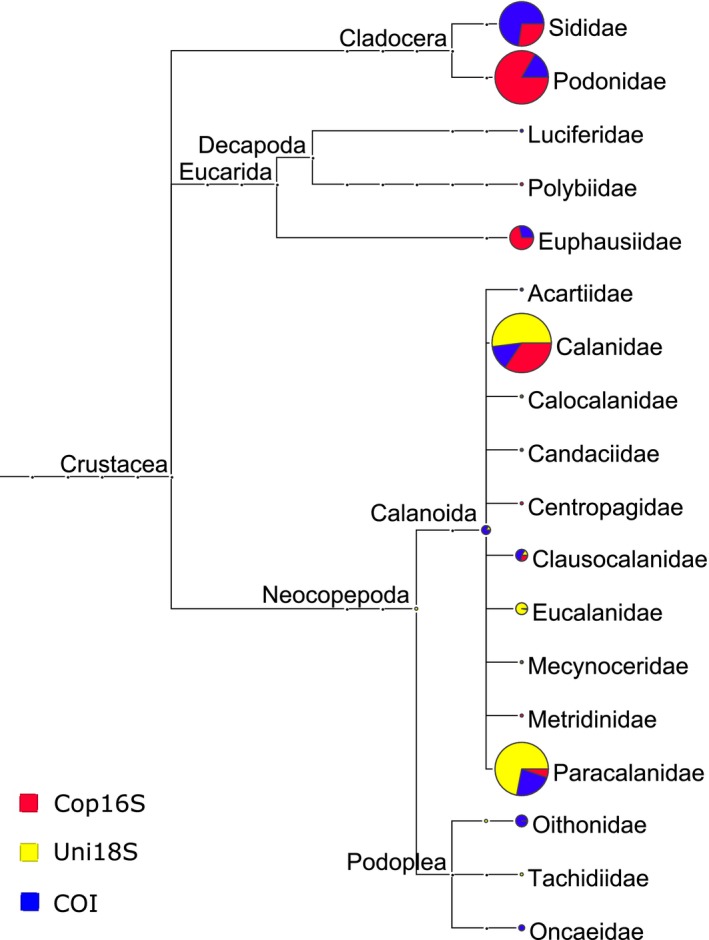
Crustacean families detected with three metabarcoding markers in zooplankton samples from site 2 in Storm Bay, Tasmania. Circle size is proportional to the number of reads assigned to that taxon based on normalized read counts

The majority of OTUs with taxonomy for each marker were assigned to metazoa (100% for Cop16S), whereas Uni18S OTUs were also assigned to Alveolata and Rhizaria, with COI OTUs assigned to these groups (except Rhizaria) as well as bacteria, Haptophyceae, fungi, stramenopiles, and Viridiplantae. Cop16S detected fewer zooplankton phyla (5) compared to COI and Uni18S (9 and 10, respectively, Table 3). However, COI detected a greater number of metazoan phyla (9) compared to Cop16S and Uni18S (5 and 7, respectively, Figure S2). Reflecting the better resolution of mitochondrial markers, COI resolved threefold more zooplankton taxa to species compared to Uni18S. Although all markers were capable of amplifying crustacean taxa, mitochondrial markers resolved three‐ to fivefold more crustacean taxa to species compared to Uni18S (Table 3). Uni18S failed to detect any cladocerans, euphausiids, or decapods, despite these taxa contributing a significant proportion of Cop16S and COI reads (Figure 3), as well as the morphology‐based counts. A much higher proportion of OTUs were not assigned taxonomy for the mitochondrial markers (*ca*. 33% vs. 2.1% for Uni18S, Table 3), likely reflecting their greater variability compared to 18S rDNA, and a less complete database for mitochondrial 16S.

#### 3.3.2 Morphology versus DNA‐based ID

**Figure 4:**
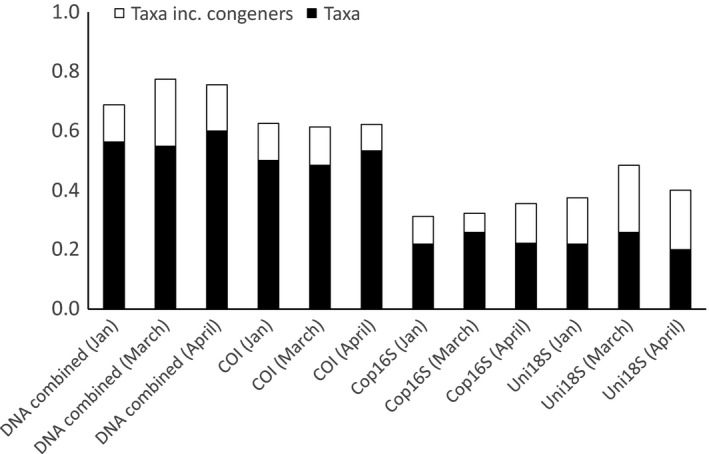
Proportion of morphologically identified zooplankton taxa from Storm Bay, Tasmania, detected using three genetic markers. Samples were collected in January, March, and April 2015

Zooplankton from site 2 were identified by morphology to species where possible, although some specimens were only assigned to phylum (e.g., larval bryozoans, undifferentiated Chaetognatha). A total of 56 zooplankton taxa (January—32, March—31, April—45, Figure 2) representing 10 phyla were identified, with more than half the taxa belonging to Copepoda (62.5%). The three genetic markers combined detected 55–60% of morphologically identified taxa at site 2 (Figure 4). DNA‐based ID would often detect congeneric species to those identified using morphology. Including congeneric species increased the proportion of taxa detected using DNA to 69–77% (Figure 4). Overall, 20 of the 25 instances where taxa (including congeners) were not detected with any marker were taxa that represented less than 2% of the total count (Table S1). The COI marker detected the greatest proportion of morphologically identified taxa (excluding congeners, 48–53%), whereas Cop16S and Uni18S detected 20–26%. The proportion of morphologically identified taxa detected by each marker was similar when restricted to crustacean taxa (Table S2). The DNA‐based approach identified 28–55 taxa (genera or higher taxonomic level) each month not detected with morphology. Combining the two methods thus increased the number of taxa detected by 88–177% compared to using morphology alone. Many of the additional taxa were unicellular (e.g., bacteria, dinoflagellates, diatoms) or algal taxa. However, the three metabarcoding markers also detected additional zooplankton taxa known to occur in Storm Bay (e.g., *Oncaea* sp., *Lucifer* sp.), or resolved morphological identifications to higher taxonomic levels (e.g., the bryozoan *Membranipora membranacea*).

#### 3.3.3 Beta‐diversity

**Figure 5:**
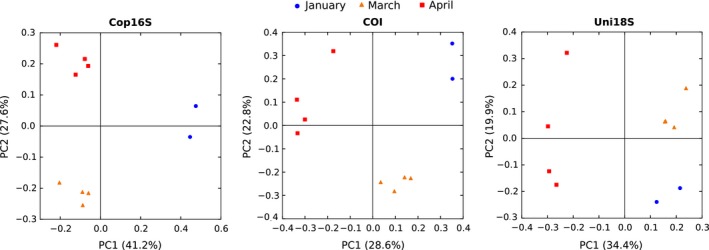
Principle coordinate analysis (PCoA) plots using Bray–Curtis distance of zooplankton communities from Storm Bay, Tasmania, derived using the metabarcoding markers Cop16S, COI, and Uni18S

Principle coordinate analysis plots showed samples clustered by month for each marker (*p *<* *.001), with greater than 48% of variation explained by collection month in each case (Uni18S: *R* ^2^ = .48, COI: *R* ^2^ = .49, Cop16S: *R* ^2^ = .67, Figure 5). The results of the SIMPER analyses showed there was typically better agreement between the morphological ID and mitochondrial markers (Table 4; Tables S3 and S4). For example, COI, Cop16S, and morphology all indicated the cladocerans *Penilia* spp. and *Podon intermedius* (absent in January and abundant reads/count in March), and the euphausiid *Nyctiphanes australis* (more abundant in January) were the main taxa behind the difference between the January and March samples. In contrast, Uni18S identified *Oikopleura dioica* (two OTUs) and several copepods as the most significant contributors to the difference between January and March (Table 4).

**Table 4:** Results of SIMPER analysis comparing January and March zooplankton samples identified using either morphology or three metabarcoding markers. The top five contributors for each method or marker are shown

Taxon / OTU	Assigned taxonomy	Contribution	*SD*	Ratio	Jan	March	Cumulative sum (%)	*p*‐Value
*Podon intermedius*		0.029	–	–	0	3.05	8.0	–
*Penilia* spp.		0.022	–	–	0	2.32	14.1	–
Paracalanidae		0.019	–	–	2.00	0	19.3	–
*Nyctiphanes australis*		0.017	–	–	2.77	1.00	24.0	–
Bivalve		0.016	–	–	1.63	0	28.2	–
Cop16S								
OTU_1	*Podon intermedius*	0.072	0.008	9.21	0	9.37	11.2	0.001
OTU_3	*Nyctiphanes australis*	0.063	0.006	10.17	8.11	0	21.0	0.005
OTU_4	*Penilia* sp.	0.049	0.009	5.46	0	6.27	28.5	0.002
OTU_2	*Calanus* sp.	0.040	0.014	2.95	7.07	1.97	34.7	0.104
OTU_6	Not assigned	0.035	0.011	3.35	5.73	1.24	40.2	0.024
COI								
OTU_1	*Penilia avirostris*	0.036	0.009	4.21	0	6.66	5.1	0.001
OTU_3	*Platycephalus richardsoni*	0.031	0.012	2.67	5.95	0.33	9.5	0.005
OTU_8	*Nyctiphanes australis*	0.028	0.006	4.51	5.24	0	13.5	0.004
OTU_2	*Podon intermedius*	0.025	0.004	5.93	0	4.80	16.9	0.003
OTU_26	*Clausocalanus ingens*	0.018	0.006	3.18	3.35	0	19.5	0.005
Uni18S								
OTU_3	*Oikopleura dioica*	0.026	0.016	1.65	7.64	3.45	5.3	0.115
OTU_2	Calanidae	0.025	0.015	1.69	7.81	3.77	10.4	0.278
OTU_8	*Ophiurida*	0.018	0.009	2.05	0.66	3.76	14.3	0.109
OTU_1	*Paracalanus* sp.	0.016	0.005	2.94	6.37	9.05	17.6	0.715
OTU_15	Neocopepoda	0.015	0.006	2.43	3.40	0.87	20.7	0.032

#### 3.3.4 Correlation of calanoid percentage biomass & HTS read counts

**Figure 6:**
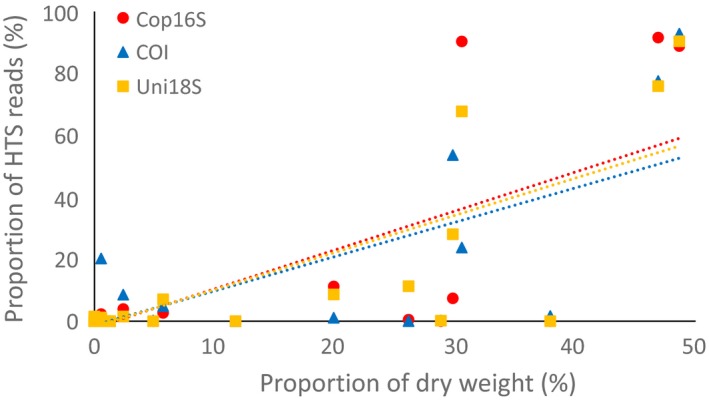
Correlation between proportion of calanoid copepod family biomass and high‐throughput sequencing reads for Cop16S (*r *=* *0.73), COI (*r *=* *0.75) and Uni18S (*r *=* *0.79) for zooplankton samples from Storm Bay, Tasmania. Data from the three months were combined

We found a positive relationship between calanoid copepod family‐level proportions of HTS reads and dry weight for each marker (Figure 6), with one or both Pearson and Spearman rank correlations significant for each marker in each month. Both correlation coefficients tended to be highest in each month for Uni18S (*r *=* *.63–.91, ρ = .43–.81, Table 5), although the Spearman's rank correlation was highest for COI in January (ρ = .87).

**Table 5:** Pearson and Spearman rank correlation coefficients between proportion of calanoid copepod family biomass and high‐throughput sequencing reads for three metabarcoding markers. *p*‐values are shown in brackets

	January		March		April	
	*r*	ρ	*r*	ρ	*r*	ρ
Cop16S	.51 (.092)	.62 (.032)	.83 (<.001)	.63 (.028)	.85 (<.001)	.31 (.33)
COI	.58 (.049)	.87 (<.001)	.81 (.002)	.80 (.002)	.84 (<.001)	.36 (.26)
Uni18S	.63 (.029)	.66 (.018)	.83 (<.001)	.81 (.001)	.91 (<.001)	.43 (.17)

#### 3.3.5 Ecological insights provided by metabarcoding

Fish eggs and larvae were present in zooplankton samples from site 2 each month, but could not be identified further by morphology. In contrast, three fish species known to be present in Storm Bay were detected with COI at site 2 over the three collection times (*Acanthaluteres vittiger*,* Aldrichetta forsteri*, and *Platycephalus richardsoni*). The additional taxonomic resolution afforded by a metabarcoding approach could thus provide valuable information on the reproductive biology of important commercial and recreational fish species.

Metabarcoding also detected taxa known to be invasive in Storm Bay (e.g., the New Zealand screwshell, *Maoricolpus roseus*, Cop16S), as well as taxa experiencing range expansions as a result of regional increases in sea surface temperature, for example, *Noctiluca scintillans* (Hallegraeff, Hosja, Knuckey, & Wilkinson, 2008). *Noctiluca scintillans* was not detected at site 2 in January or March, but was present in all four replicates for both COI and Uni18S in April, immediately prior to a *Noctiluca* bloom at sites around Storm Bay in May 2015 (personal observation). Crustacean parasites of the Syndinidae family (*Syndinium turbo* (Uni18S) and *Hematodinium* (COI)) were detected in all samples.

Blue whale (*Balaenoptera musculus*) was detected with COI in the two January site 2 PCR replicates in both HTS runs. Genomic blue whale DNA is present in our laboratory, and hence, detection could represent contamination. However, blue whale was not detected in no template controls and the species was sighted near Storm Bay off the Tasman Peninsula in February 2015 (http://wildoceantasmania.com.au/blue-whale-sighting/), suggesting the detection is plausible.

## 4 Discussion

The aim of this study was to compare the performance of metabarcoding markers targeting either nuclear (18S) or mitochondrial (16S or COI) DNA for characterizing zooplankton communities in terms of taxonomic coverage and resolution, correspondence with morphology‐based identification, and their ability to quantify relative abundance. We also explored the reproducibility and impact of thermal cycling protocol on OTU and taxon detection for COI.

The increased number of OTUs and slightly greater number of taxa detected using a single low annealing temperature compared to the touchdown protocol for COI demonstrates the importance of considering thermal cycling protocols in metabarcoding studies. The detection of additional taxa supports the use of low annealing temperatures to maximize taxonomic coverage for any given marker (Clarke et al., 2014; Sipos et al., 2007). Amplification and sequencing of COI amplicons generated using the low annealing temperature protocol was highly repeatable in spite of using different sequencing chemistries (v2 and v3) and number of sequencing cycles (2 × 250 and 2 × 300), with OTUs representing >0.1% of reads in one replicate almost always detected in the corresponding replicate. Estimating the reproducibility of rare OTUs by sequencing a set of technical replicates in each study could provide a means to establish an abundance threshold for OTU retention, as nonreproducible OTUs are more likely to represent PCR or sequencing artifacts (De Barba et al., 2014; Ficetola et al., 2015).

The use of different polymerases for COI (AmpliTaq Gold) and 16S/18S (Phusion) complicates the marker comparison in this study. Phusion polymerase could not be used for the COI marker due to inosine residues in one primer. The use of a nonproofreading polymerase could inflate the number of OTUs detected for COI. Similarly, using a proofreading polymerase could explain the reduced number of OTUs detected with 18S in this study compared to other zooplankton metabarcoding studies (Brown et al., 2016; Chain et al., 2016). However, we feel comparisons of the number of taxa detected with each marker are robust.

Many of the results of this study are consistent with well‐recognized characteristics of 18S and mitochondrial markers identified in previous studies. As per Tang et al. (2012), we find 18S provided poor species resolution compared to COI and 16S mitochondrial markers, despite targeting a longer variable region (V4) than used in many studies (e.g., Jarman et al., 2013). The taxonomic coverage reflected the target taxa for each marker to some degree, with Uni18S detecting one more zooplankton phylum than COI (Table 3), but COI detecting more metazoan phyla. In contrast to the findings of Zhan et al. (2014), the mitochondrial 16S marker also provided slightly better taxonomic coverage than Uni18S within the crustacea (four vs. three orders, 11 vs. 10 families). Although direct comparison is complicated given the use of different DNA polymerases, the Cop16S primers and those used by Zhan et al. (2014) bind to almost identical sites in the mitochondrial 16S rDNA. The lower annealing temperature used in this study (45°C vs. 50°C) may have contributed to the broader coverage of the Cop16S marker observed. As highlighted in many metabarcoding studies to date, all three markers suffered from incomplete reference databases, with 2.1–33.7% of OTUs unassigned for each marker, and many OTUs assigned to congeneric species of those identified using morphology.

All three metabarcoding markers revealed that distinct zooplankton communities were present at the three time points (Figure 5), hence are all potentially valuable tools for monitoring seasonal and temporal change. However, the markers differed in the taxa identified as driving the difference between months. For example, COI and Cop16S identified the cladocerans *Penilia* spp. and *Podon intermedius*, and the euphausiid *Nyctiphanes australis* as the key taxa driving the difference between January and March samples (Table 4), whereas Uni18S failed to detect these taxa in any month (Figure 3). It is unclear why Uni18S failed to detect euphausiids and cladocerans, despite their high proportional representation in both the morphology‐based counts and mitochondrial HTS reads. Although 18S sequences were not available for *Nyctiphanes australis* or *Podon intermedius*, no primer‐template mismatches were identified in the available sequence data for *Penilia avirostris*, or congeneric *Podon* or *Nyctiphanes* species. However, the predicted Uni18S amplicon for *Penilia avirostris* was more than 100 bp longer than the mean length of the Uni18S OTUs in this study (419 ± 26 bp, mean ± *SD*). Amplicon length polymorphism has been shown to cause differential amplification and taxonomic bias in bacterial and fungal HTS studies (Ihrmark et al., 2012; Ziesemer et al., 2015) and may explain the failure to detect cladocerans with Uni18S. Predicted Uni18S amplicon lengths for Euphausiidae other than *Nyctiphanes australis* are close to the mean length observed in this study (431 bp), and thus, it remains unclear why euphausiids were not detected with 18S. If we had only analyzed our samples with 18S instead of three metabarcoding markers and morphology, we would have failed to detect the important contribution of the cladocerans and euphausiids to the beta‐diversity pattern observed, highlighting the benefit of using multiple markers or approaches for biodiversity assessments.

Several metabarcoding studies have identified a positive relationship between biomass and the number of HTS reads for a given taxon for both nuclear rDNA and mitochondrial markers (e.g., Evans et al., 2016; Sun et al., 2015). In this study, we examined the relationship for calanoid copepods as all markers were capable of PCR‐amplifying this group, and we could estimate biomass from morphology‐based counts using the approach of Hirai et al. (2015). We found all three markers displayed a positive relationship between biomass and HTS reads for calanoid families. Interestingly, both Pearson and Spearman rank correlations were typically strongest for the nuclear 18S rDNA marker. Many studies have shown strong correlations between biovolume and 18S copy number for a range of eukaryotic taxa, including metazoans (Godhe et al., 2008; de Vargas et al., 2015; Zhu, Massana, Not, Marie, & Vaulot, 2005). Several factors could reduce the strength of the correlation between biomass and HTS reads in this study, including using separate samples for DNA‐based and morphology‐based identification, and estimating biomass using conversion factors rather than direct measurement. Our results suggest the number of 18S rDNA HTS reads provides a better proxy for calanoid copepod biomass than mitochondrial markers, but should be confirmed and extended to other zooplankton groups using well‐characterized samples such as mock communities.

## 5 Conclusion

Our study extends previous research demonstrating the value of metabarcoding for rapidly surveying biodiversity, including the potential to identify nonmetazoans and developmental stages such as eggs and larvae, as well as environmental DNA (whale) and parasite detections not possible with traditional methods. It is worth noting that additional data on developmental stage or sex can be obtained using morphology that is not available using a metabarcoding approach on its own. Our results show that different metabarcoding markers and/or protocols provide slightly different views of genetic biodiversity. Comparisons with morphology‐based datasets are useful for showing which markers best match traditional datasets and highlighting potential shortcomings of markers. Standardization of thermal cycling protocols and markers will be required to allow valid comparisons between studies.

In contrast to previous studies that recommend 18S as the most suitable marker for surveying zooplankton communities (Zhan et al., 2014), we find COI provided similar coverage of zooplankton phyla, but better taxonomic resolution (Table 3) and agreement between morphology‐ and DNA‐based identifications (Figure 4). Although the use of COI for metabarcoding has been questioned due to lack of conserved primer‐binding sites (Clarke et al., 2014; Deagle et al., 2014), the taxonomic coverage and resolution provided by degenerate COI primers, combined with a comparatively well‐developed reference sequence database, make them valuable metabarcoding markers for biodiversity assessment. The potential for retrieving at least semiquantitative abundance data was confirmed for all markers, with 18S providing the strongest relationship between calanoid copepod biomass and number of HTS reads. However, alternatives to PCR‐based approaches may be required to accurately quantify species abundance with high‐throughput sequencing (Dowle, Pochon, Banks, Shearer, & Wood, 2015; Zhou et al., 2013).

## Data Accessibility

High‐throughput sequencing reads (FASTQ) for the two MiSeq libraries and the OTU tables for each locus are available on the Dryad Digital Repository, doi:10.5061/dryad.gf246.

## Conflict of Interest

None declared.
