# Supplementary material for: Effect of marker choice and thermal cycling protocol on zooplankton DNA metabarcoding studies
Source: Ecol Evol. 2017 Jan 12;7(3):873–83. doi: 10.1002/ece3.2667 (PMC5288259; doi:10.1002/ece3.2667)
Supplement: Supplementary file 1 [file ECE3-7-873-s001.docx]

**Appendix**

**Table S1.** Morphology-based zooplankton counts for Storm Bay (site 2), Tasmania. Samples were collected between January and April, 2015. Instances where none of the three DNA markers detected a taxon (or congeneric species) in a given month are shown in red, those representing greater than 2% of the total count for the month are underlined.

| **Taxon** | **Jan** | **March** | **April** |
| --- | --- | --- | --- |
| Acartia - undifferentiated | 2 | 2 | 5 |
| Acartia danae | 1 | 1 | 1 |
| Acartia tranteri | 55 | 21 | 10 |
| Bivalve - undifferentiated | 7 | 0 | 60 |
| Calanidae - undifferentiated | 14 | 1 | 12 |
| Calanoides spp. | 2 | 0 | 3 |
| Calanus australis | 5 | 4 | 23 |
| Calocalanus pavo | 0 | 0 | 1 |
| Calocalanus plumulosus | 2 | 0 | 0 |
| Calocalanus styliremis | 1 | 1 | 0 |
| Centropages australiensis | 0 | 1 | 0 |
| Centropages bradyi | 0 | 0 | 1 |
| Class Holothuroidea larvae - undifferentiated | 73 | 46 | 3 |
| Clausocalanidae - undifferentiated | 1 | 0 | 0 |
| Clausocalanus - undifferentiated | 6 | 0 | 2 |
| Clausocalanus jobei | 0 | 0 | 13 |
| Clausocalanus pergens | 3 | 0 | 1 |
| Crustacean nauplii | 26 | 2 | 35 |
| Ctenocalanus vanus | 0 | 0 | 4 |
| Cyphonaute larvae | 8 | 4 | 15 |
| Decapod larvae | 0 | 0 | 2 |
| Doliolum - undifferentiated | 0 | 0 | 4 |
| Eucalanus elongatus | 0 | 0 | 1 |
| Euterpina acutifrons | 0 | 0 | 2 |
| Evadne spp. | 0 | 1 | 110 |
| Farranula rostrata | 0 | 0 | 2 |
| Fish eggs | 22 | 2 | 34 |
| Foraminifera | 2 | 8 | 5 |
| Fritillariidae - undifferentiated | 0 | 0 | 65 |
| Hydromedusae indet | 1 | 3 | 8 |
| Larval fish | 0 | 1 | 0 |
| Lucifer hanseni | 0 | 7 | 0 |
| Mecynocera clausi | 1 | 0 | 2 |
| Mesosagitta minima | 0 | 0 | 8 |
| Nannocalanus minor | 0 | 1 | 0 |
| Nyctiphanes australis | 59 | 1 | 2 |
| Oculosetella gracilis | 1 | 1 | 0 |
| Oikopleuridae - undifferentiated | 82 | 37 | 28 |
| Oithona atlantica | 29 | 10 | 14 |
| Oithona longispina | 0 | 1 | 0 |
| Oithona setigera | 0 | 0 | 4 |
| Oithona similis | 79 | 24 | 64 |
| Oithonidae - undifferentiated | 15 | 8 | 16 |
| Oncaea - undifferentiated | 1 | 0 | 3 |

**Table S1.** Morphology-based zooplankton counts (continued).

| **Taxon** | **Jan** | **March** | **April** |
| --- | --- | --- | --- |
| Oncaea media | 0 | 0 | 3 |
| Paracalanidae - undifferentiated | 16 | 0 | 4 |
| Paracalanus indicus | 171 | 136 | 56 |
| Penilia spp. | 0 | 29 | 47 |
| Phylum Chaetognatha - undifferentiated | 0 | 2 | 0 |
| Phylum Echinodermata - undifferentiated | 0 | 4 | 1 |
| Podon intermedius | 0 | 86 | 18 |
| Polychaeta-pelagic-undifferentiated | 1 | 0 | 0 |
| Pteropoda indet | 59 | 20 | 13 |
| Serratosagitta spp. | 7 | 0 | 0 |
| Subeucalanus pileatus | 0 | 0 | 9 |
| Temora turbinata | 5 | 5 | 13 |
| Total count | 757 | 470 | 728 |

**Table S2.** Proportion of morphologically identified crustacean taxa from Storm Bay, Tasmania, detected using three genetic markers. Samples were collected in January, March and April 2015.

|  | **January** | **March** | **April** |
| --- | --- | --- | --- |
| LerayCOI | 12/22 (55%) | 9/21 (43%) | 19/33 (58%) |
| LerayCOI, inc. congeners | 16/22 (73%) | 13/21 (62%) | 23/33 (70%) |
| Cop16S | 6/22 (27%) | 5/21 (24%) | 8/33 (24%) |
| Cop16S, inc. congeners | 9/22 (41%) | 7/21 (33%) | 14/33 (42%) |
| Uni18S | 6/22 (27%) | 5/21 (24%) | 8/33 (24%) |
| Uni18S, inc. congeners | 11/22 (50%) | 12/21 (57%) | 17/33 (58%) |

**Table S3.** Results of SIMPER analysis for January/April zooplankton samples identified using either morphology or three metabarcoding markers. The top five contributors for each method or marker are shown.

| **Taxon / OTU** | **Assigned taxonomy** | **Contribution** | **SD** | **Ratio** | **Jan** | **April** | **Cumulative sum (%)** | ***P*-value** |
| --- | --- | --- | --- | --- | --- | --- | --- | --- |
| *Evadne* spp. |  | 0.024 | - | - | 0 | 3.24 | 6.42 | - |
| Fritillariidae |  | 0.021 | - | - | 0 | 2.84 | 12.06 | - |
| *Penilia* spp. |  | 0.020 | - | - | 0 | 2.62 | 17.25 | - |
| *Podon intermedius* |  | 0.015 | - | - | 0 | 2.06 | 21.33 | - |
| *Clausocalanus jobei* |  | 0.014 | - | - | 0 | 1.90 | 25.10 | - |
|  |  |  |  |  |  |  |  |  |
| **Cop16S** |  |  |  |  |  |  |  |  |
| OTU_3 | *Nyctiphanes australis* | 0.060 | 0.006 | 10.14 | 8.11 | 0 | 8.95 | 0.020 |
| OTU_6 | Not assigned | 0.043 | 0.011 | 4.03 | 5.73 | 0 | 15.31 | 0.001 |
| OTU_7 | *Maoricolpus roseus* | 0.040 | 0.003 | 11.78 | 5.33 | 0 | 21.18 | 0.015 |
| OTU_4 | *Penilia* sp. | 0.039 | 0.008 | 4.68 | 0 | 5.29 | 27.03 | 0.023 |
| OTU_1 | *Podon intermedius* | 0.036 | 0.013 | 2.73 | 0 | 4.73 | 32.32 | 0.603 |
|  |  |  |  |  |  |  |  |  |
| **COI** |  |  |  |  |  |  |  |  |
| OTU_3 | *Platycephalus richardsoni* | 0.029 | 0.008 | 3.50 | 5.95 | 0 | 3.75 | 0.015 |
| OTU_8 | *Nyctiphanes australis* | 0.024 | 0.006 | 4.20 | 5.24 | 0.33 | 6.81 | 0.016 |
| OTU_1 | *Penilia avirostris* | 0.023 | 0.005 | 4.77 | 0 | 5.03 | 9.83 | 0.060 |
| OTU_4 | *Podosira stelligera* | 0.019 | 0.003 | 5.84 | 0 | 4.06 | 12.28 | 0.020 |
| OTU_28 | Not assigned | 0.017 | 0.004 | 4.09 | 3.48 | 0 | 14.47 | 0.005 |
|  |  |  |  |  |  |  |  |  |
| **Uni18S** |  |  |  |  |  |  |  |  |
| OTU_3 | *Oikopleura dioica* | 0.034 | 0.006 | 5.70 | 7.64 | 2.16 | 5.88 | 0.027 |
| OTU_4 | Eucalanidae | 0.025 | 0.011 | 2.28 | 0.00 | 4.19 | 10.27 | 0.048 |
| OTU_15 | Neocopepoda | 0.021 | 0.005 | 4.64 | 3.40 | 0.00 | 13.90 | 0.003 |
| OTU_51 | *Oikopleura dioica* | 0.019 | 0.004 | 4.95 | 3.74 | 0.60 | 17.24 | 0.011 |
| OTU_6 | *Noctiluca scintillans* | 0.019 | 0.015 | 1.25 | 0.00 | 3.15 | 20.55 | 0.195 |

**Table S4.** Results of SIMPER analysis for March/April zooplankton samples identified using either morphology or three metabarcoding markers. The top five contributors for each method or marker are shown.

| **Taxon / OTU** | **Assigned taxonomy** | **Contribution** | **SD** | **Ratio** | **March** | **April** | **Cumulative sum (%)** | ***P*-value** |
| --- | --- | --- | --- | --- | --- | --- | --- | --- |
| Fritillariidae |  | 0.023 | - | - | 0 | 2.84 | 5.32 | - |
| Bivalve |  | 0.022 | - | - | 0 | 2.78 | 10.54 | - |
| *Evadne* spp. |  | 0.018 | - | - | 1 | 3.24 | 14.74 | - |
| *Clausocalanus jobei* |  | 0.015 | - | - | 0 | 1.90 | 18.30 | - |
| *Subeucalanus pileatus* |  | 0.014 | - | - | 0 | 1.73 | 21.55 | - |
|  |  |  |  |  |  |  |  |  |
| **Cop16S** |  |  |  |  |  |  |  |  |
| OTU_2 | *Calanus* sp. | 0.046 | 0.025 | 1.86 | 1.97 | 7.03 | 8.17 | 0.015 |
| OTU_5 | *Pseudevadne tergestina* | 0.043 | 0.018 | 2.36 | 0 | 4.71 | 15.77 | 0.001 |
| OTU_1 | *Podon intermedius* | 0.040 | 0.015 | 2.61 | 9.37 | 4.73 | 22.85 | 0.308 |
| OTU_13 | *Clausocalanus* sp. AR-2011 | 0.029 | 0.008 | 3.66 | 0 | 3.26 | 27.96 | 0.001 |
| OTU_14 | *Paracalanus* sp. | 0.024 | 0.012 | 2.04 | 2.93 | 0.25 | 32.15 | 0.001 |
|  |  |  |  |  |  |  |  |  |
| **COI** |  |  |  |  |  |  |  |  |
| OTU_6 | *Calanus australis* | 0.020 | 0.012 | 1.67 | 1.05 | 4.82 | 2.87 | 0.008 |
| OTU_11 | *Paracalanus indicus* | 0.020 | 0.009 | 2.19 | 4.41 | 0.58 | 5.69 | 0.002 |
| OTU_4 | *Podosira stelligera* | 0.017 | 0.006 | 2.76 | 0.59 | 4.06 | 8.13 | 0.006 |
| OTU_2 | *Podon intermedius* | 0.014 | 0.008 | 1.72 | 4.80 | 1.87 | 10.16 | 0.292 |
| OTU_16 | Eukaryota | 0.014 | 0.003 | 4.29 | 0 | 2.84 | 12.16 | 0.004 |
|  |  |  |  |  |  |  |  |  |
| **Uni18S** |  |  |  |  |  |  |  |  |
| OTU_1 | *Paracalanus* | 0.031 | 0.012 | 2.58 | 9.05 | 4.14 | 5.09 | 0.003 |
| OTU_2 | Calanidae | 0.028 | 0.018 | 1.54 | 3.77 | 7.62 | 9.66 | 0.041 |
| OTU_4 | Eucalanidae | 0.025 | 0.011 | 2.31 | 0.00 | 4.19 | 13.83 | 0.010 |
| OTU_8 | *Ophiurida* | 0.023 | 0.009 | 2.73 | 3.76 | 0.00 | 17.67 | 0.003 |
| OTU_6 | *Noctiluca scintillans* | 0.019 | 0.015 | 1.28 | 0.00 | 3.15 | 20.81 | 0.010 |

**Figure S1.** Rarefaction curves for number of OTUs per sample against sequencing depth for the three metabarcoding markers. Values are means ± SD.

**Figure S2.** Phyla detected with three metabarcoding markers in zooplankton samples from site 2 in Storm Bay, Tasmania. Some non-zooplankton groups were collapsed for clarity. Circle size is proportional to the number of reads (log-scale) assigned to that taxon based on normalized read counts.
